# The potential role of regenerative medicine in the management of traumatic patients

**DOI:** 10.5249/jivr.v7i1.704

**Published:** 2015-01

**Authors:** Mahmoudreza Moradi, Brandy Hood, Marzieh Moradi, Anthony Atala

**Affiliations:** ^a^Division of Regenerative Medicine, Fertility-Infertility Research Center, Kermanshah University of Medical Science, Kermanshah, Iran.; ^*b*^Department of Urology, Wake Forest Institute for Regenerative Medicine, Wake Forest School of Medicine, Winston-Salem, NC,USA.

**Keywords:** Trauma, Tissue Engineering, Regenerative medicine

## Abstract

Traumatic injury represents the most common cause of death in ages 1 to 44 years and a significant proportion of patients treated in hospital emergency wards each year. Unfortunately, for patients who survive their injuries, survival is not equal to complete recovery. Many traumatic injuries are difficult to treat with conventional therapy and result in permanent disability. In such situations, regenerative medicine has the potential to play an important role in recovery of function. Regenerative medicine is a field that seeks to maintain or restore function with the development of biological substitutes for diseased or damaged tissues. Several regenerative approaches are currently under investigation, with a few achieving clinical application. For example, engineered skin has gained FDA approval, and more than 20 tissue engineered skin substitutes are now commercially available. Other organ systems with promising animal models and small human series include the central and peripheral nervous systems, the musculoskeletal system, the respiratory and genitourinary tracts, and others. This paper will be a clinically oriented review of the regenerative approaches currently under investigation of special interest to those caring for traumatic patients.

## Introduction

Trauma is the most common cause of death in people between 1 and 44 years and the third most common cause of death overall. Trauma causes more than 150,000 deaths per year in the United States. It has been reported that there were more than 167,000 trauma-related deaths in the US in 2004, but more than 29.6 million trauma patients treated in emergency wards in that year.^1^ In the recent wars in Afghanistan and Iraq, significant improvements in personal protective devices have decreased the mortality rate relative to previous wars. Unfortunately, we have seen that survival, per se, does not equal complete recovery.^2^ Thus, trauma-related mortality rates are not a good indicator of the full impact of trauma on our society. The severity of many traumatic injuries makes them difficult to treat with conventional therapy.

In these situations regenerative medicine has the potential to play an important role. Regenerative medicine and tissue engineering is “an interdisciplinary field which applies the principles of engineering and life sciences towards the development of biological substitutes that aim to maintain, restore or improve tissue function” (Atala et al, 2001).^3^ As a result of the increasing human life span, the number of patients who need transplant organs has increased.

Issues with rejection and immunosuppressive drug complications have also increased the need for alternative treatments (**regenerative medicine**).^4^

This article will review the regenerative medicine approaches used in traumatic organ animal models and human studies. The discussion will be clinically oriented, and as such, a discussion about basic techniques is beyond scope of this article.

**Central Nervous System**

Previously, scientists believed that the adult central nervous system (CNS) was unable to repair itself after injury or disease^5^ because of the limiting effects of inflammation, cellular loss, cavitations and glial scarring.^6^ However, repair of the CNS is currently one of the most promising fields of regenerative medicine.^5^

Several techniques have been used to create neural tissue in animal models. Spinal cord neural progenitor cells (SCNPCs) were cultured in vitro and transplanted into rat spinal cords after spinal cord injury (SCI). At follow-up, SCNPCs survived, but there was no significant differentiation toward neural morphology or neural functional improvement.^6^ In other studies, human umbilical cord blood (hUCB) cells were used to treat neurological disorders in various animal models. These cells are able to differentiate into several other cell types, including neural cells. Also, they have the ability to produce neurotropic factors that bring about the release of several growth factors. These growth factors stimulate cell survival and angiogenesis and have anti-inflammatory effects. Currently, there are few clinical studies using hUCB because the safety and efficacy has not been determined.^7^ The role of neural supporting cells (astrocytes and oligodendrocytes) in the restoration of neurological function has also been investigated. GRP cell-derived astrocytes (GDAs) were transplanted into transected rat spinal cords. After transplantation, axonal regeneration, neural cell survival, and tissue structure realignment was observed. This study demonstrates a role for astrocyte transplantation therapy in the restoration of neurological function.^8^ In animal models of SCI, transplantation of olfactory ensheathing cells supports axonal remyelination and migration along the spinal cord. This technique is useful for cases in which the anatomy is preserved but axons are physiologically disrupted.^9^ Some substances, such as glial cell line derived neurotrophic factor (GDNF), can increase the ability of a nerve graft placed in a spinal cord transaction gap to promote motor recovery and nerve growth.^10^ Stem cells are an ideal way to treat diseases and trauma of the nervous system.

Transplantation of embryonic or adult stem cells can replace damaged cells and aid in the recovery of function.^11^ In a study by Nori et al. mouse induced pluripotent stem cells (iPSCs) and neural stem cell/progenitor cells (NS/PCs) were transplanted into the spinal cord 9 days after injury. These cells differentiate into all three neural lines and promote re-myelination, axonal re-growth and recovery of motor function. No evidence of teratoma or other tumor formation was seen.^12^ In another study, adipose-derived stromal cells were harvested from mice and humans and injected intravenously into mice with calvarial defects. These mice were compared to controls injected with saline only. Cell migration and bone formation was evaluated by histology and micro-CT scan. Histologic evidence of bone formation was seen in both mouse and human cells, but by CT analysis, only human cells promoted ossification. These results suggest that intravenous injection of human adipose-derived stromal cells may be an effective route for future skeletal regeneration.^13^ Recently, neuroepithelial-like stem cells have been developed from human iPS cells. Transplantation of these cells into a mouse model with SCI improves recovery of hind limb motor function.^14^ Studies have investigated the possible benefit of combined cell therapy in SCI models. Ban et al. used activated Schwann cells (ASCs) and bone mesenchymal stem cells (BMSCs) in rats with traumatic SCI. Co-transplantation of these cells led to recovery of hindlimb function, decreased glial scar formation and remyelination of damaged axons^15^ Also, co-transplantation of peripheral nerve tissue with fetal brain tissue has been shown to be more effective for spinal cord reconstruction after injury.^16^

Biomaterial scaffolds create an environment for directed cell growth that may eventually lead to regeneration of specific tissues or even complete organs. Cell behaviors such as migration and proliferation are facilitated by communication of cells with the micro-environment, nearby cells and with the matrix itself. Thus, scaffolds may play an important role in improving some tissue engineering-based therapies.^17^ In one human model, human mesenchymal stem cells harvested from the bone marrow and seeded with bioceramics, facilitated bone regeneration after spinal fusion. This method may be an alternative to autologous and allogenic bone fusion.^18^

In a human series with SCI (2 male and 2 female) concentrated cellular bone marrow was implanted in an intraspinal lesion at the time of surgery. After 1 year of follow up, three showed improvement. No complication was seen as a result of intralesional bone marrow cell injection. Considering these results, the potential role of regenerative medicine in the treatment of CNS lesions is no longer speculation.^19^

**Peripheral Nervous system**

The management of injury-associated peripheral nerve defects is difficult because of the absence of suitable autologous nerves for transplantation.^20^ One study has found that biomaterial gel made from proteins found in human hair promotes nerve regeneration through activation of Schwann cells. This study and others like it have demonstrated nerve regeneration results comparable to autograft nerve repair.^20^ In another similar study, the use of keratin gel was a viable guiding material for Schwann cell and axon migration and proliferation, functions that are essential for nerve repair.^21^ It has also been shown that Spidrex conduits, a silk-based biomaterial, promote axonal regeneration and functional recovery, and may be useful for clinical application.^22^

As in the CNS, the use of stem cells in both animal models^23^ and human models^24^ of peripheral nerve injury has been reported. After 60 days of follow-up umbilical cord mesenchymal stem cells injected intravenously in a patient with both nonunion and nerve injury resulted in improved nerve reflex, muscle tone and muscle strength. In addition, nerve conduction velocity increased and the fracture gap disappeared.^24^ These advances may open up exciting new avenues of treatment for patients with peripheral nervous system injury.

**Musculoskeletal system**

Autologous bone grafts are the gold standard for reconstruction of large bone defects. However, surgical stress to the portion of normal bone being extracted and the amount of extractable bone present limit this method.^25^ As in other organs, stem cell based therapies have the potential to improve the treatment of osseous defects. Osteoblasts derived from induced adipose-derived stromal cells from rabbits were seeded on chitosan-tricalcium-phosphate-gelatin (CS-TCP-Gel) and chitosan (Cs) scaffolds, that reconstructing reinforced and conventional periostea respectively. These materials were transplanted into long bone defects in rabbits. The findings of this study showed that reinforced periosteum performs better than conventional periosteum and is more suitable for repair of weight-bearing bones.^26^ A study by Levi et al. has shown that human adipose-derived stromal stem cells (ASCs) can heal acute calvarial defects in mice, but cannot repair chronic defects. This difference has been attributed to endogenous bone morphogenetic protein (BMP) activation after trauma.^27^ Thus, local delivery of BMP may enable regeneration of large bone defects without bone grafting.^28^ Also, short poly-Nacetyl glucosamine (sNAG) fibers have been shown to activate bone regeneration in rabbit femurs.^29^

Combinations of biological substance may be useful for treatment of overuse injuries. Administration of autologous platelet-rich plasma (PRP) and isolated bone marrow mononucleated cells (BMMNCs) in competition horses showed significant improvement of lameness and return to competitive status. This suggests that similar application of appropriate cells and growth factors (GFs) may be useful for treatment of human athletes.^30^ Combinations may have other applications as well. Simultaneous sustained release of an antibiotic and a human growth factor is one viable option for decreasing infection rates and improving vascularization and healing of bone grafts in contaminated open fractures. ^31^ Cryptic peptide, derived from the C-terminal telopeptide of collagen 3, is another biological substance shown to have implications in regenerative therapies. In a murine model of limb amputation, cryptic peptide caused bone nodule formation at the site of amputation. In addition, cryptic peptide has the ability to alter stem cell recruitment and differentiation at the injury site, and may serve as a new method for influencing stem cells in that local environment.^32^ In one human case report, decellularized bovine trabecular bone was seeded with autologous bone marrow cells, cultured for three weeks, and subsequently implanted into 72mm distal tibial defect and fixed with an intramedullary nail. After six weeks, bone formation was seen around the graft, and the patient recovered the ability to walk. At two years no complications have occurred. This graft represents a useful alternative for long bone defect repair.^33^

Repair of articular cartilage is difficult, as it is both avascular and aneural.^34^ Recently tissue engineering of cartilage has entered into clinical practice.^35^ Autologous chondrocytes were recently implanted into articular cartilage defects in multiple human series. After long-term follow-up, results were excellent with patients returning to normal activity levels.^36,37,38^ Hyalouronan-based scaffolds seeded with autologous chondrocytes have been implanted in the knees of 53 patients with up to 7 years follow up. Hyalograft C autografts resulted in clinical improvement in young healthy patients with single cartilage defects, but results were poor in patients with osteoarthritis.^39^ Autologous mesenchymal stem cells harvested from the iliac crest and cultured with growth factors have also been implanted into an adult human knee, resulting in an increase in meniscal cartilage volume.^40^

Damaged tendons rarely heal completely. Recently, regenerative medicine has made complete repair possible by means of an extracellular matrix derived from porcine small intestinal submucosa. This technique may aid in the organization of host tissue^41^ and allow tendon regeneration with stem cells.^42,43^ Three hundred British orthopedic surgeons specializing in knee surgery were invited to participate in an online questionnaire about tissue engineering of the anterior cruciate ligament. Overall, most surgeons would be prepared to use this technique if it were an improvement over the current technique.^44^

**Skin**

Regenerative science has rapidly improved the clinical management of wounds. Currently there are more than 20 skin substitutes commercially available. These may contain allogenic or autogenic living cells or no living cells.^45^ The first product commercially available was an autologous epidermal substitute.^46^ Later, Kuroyanagi developed an allogenic cultured dermal substitute. This substitute consists of a 2-layered spongy matrix of hyaluronic acid and atelocollagen containing fibroblasts. The skin substitute releases numerous substances necessary for wound healing.^46^ Platelet–derived growth factor is one substance necessary for wound repair, participating in chemotaxis, cell proliferation, etc.^47,48^ Topical therapy with platelet gel and PG-derived GF also has a potential role in wound healing.^48^ Tissue engineered skin has now been approved by the United States Food and Drug Administration (FDA). In some human reports, tissue engineered skin was used to treat dermal atrophy secondary to traumatic avulsion^49,50^ and for the treatment of wounds in the premature neonate in order to prevent hypertonic wound extravasations.^51^

Use of stem cells for the treatment of skin damage has been reported. Adipose-derived stem cells^52,53^ and bone marrow-derived stem cells ^54^ with or without dermal substitute were used in animal and human model with positive results. Adipose-derived stem cells exhibit antioxidant effects, increase vascularity and collagen synthesis and improve skin regeneration. ^52,53^ These cells can usually still be found in deep layers of skin wounds in patients with major traumatic injuries, such as extensive burns. Thus, adipose-derived stem cells may be obtained from discarded skin from burn victims. ^55^ Application of stem cells for the management of radiation burns has been reported in animal models via systemic transplantation ^56^ and local cell therapy in human cases.^57,58^ Such therapies may provide new hope in the treatment of radiation burns and radiotherapy complications.

Delayed wound healing (two weeks after the injury) is usually associated with poor aesthetics and impaired function. In order to eliminate the need for cell culture before treatment, an innovative cell isolation technique has been used in which a biopsy of healthy skin is exposed to two enzymes and the desired cells transferred to ringer’s lactate solution. After wound debridement, the cells are immediately transplanted with a spraying mechanism.^59,60^

Another novel method for improving wound healing is the application of an active dressing that supplies cell support and provokes regeneration by wound irrigation.^60^

**Respiratory Tract**

Lung injuries can cause significant mortality and morbidity.^61^ There are several sources of adult stem cells in respiratory tract, which contribute to repair of the respiratory tract lining. To date, great progress has been made in the field of tracheal bioengineering. One method employed biodegradable three-dimensional scaffolds for cell attachment, cell differentiation and extracellular matrix production and finally whole tracheal regeneration. Using this method the world’s first bioengineered trachea was produced.^62^

**Genitourinary system**

Traumatic events can lead to organ damage or even loss within the genitourinary tract. Several regenerative medicine strategies for replacing these organs are currently under investigation.^63^ Renal tissue is one the most difficult to regenerate and most efforts for kidney regeneration have focused on cell therapy.^3^ In one study, culture of primary renal cells in three-dimensional collagen-based scaffolds promoted regeneration of tubule and glomeruluslike structures that had positive staining for Tamm-Horsfall protein.^64^ Unfortunately, due to the complexity of both the anatomy and function of the kidney, regenerative medicine does not yet play a prominent role in the treatment of patients with traumatic renal injuries.

Regeneration of the ureter has been performed in animal models at several centers. Use of small-intestinal sub-mucosal as a ureteral replacement material^65^ and transplantation of urothelial and smooth cells on tubular polymer scaffolds^66^ have both been done in canine models with good results. However, the relatively small incidence of ureteral injury and the large expense involved in ureteral regeneration currently limit the translation of these techniques to humans.^3^

A number of animal studies and human clinical experiences have shown the value of bladder tissue engineering.^67^ Urothelial progenitor cells can be harvested from the bladder, especially the bladder neck and trigone areas.^3^ Additionally, amniotic-derived, bone marrow-derived and urine-derived stem cells have the ability to differentiate into bladder tissue.^3,68^ Still, the use of biomaterial-based scaffolds seeded with autologous urothelial and smooth muscle cells is the most efficacious method for engineering bladder tissue.^67^ Using this method Atala et al. constructed neobladders for seven patients with myelomeningocele. After 22-61 months of follow-up biopsies of the neobladders showed normal structure and phenotype.^69^

Various methods have been proposed for urethral regeneration.^3^ Acellular collagen matrices harvested from bladder submucosa have been used experimentally and clinically for onlay urethral substitution with good results.^70,71^ More than 200 patients with urethral stricture have been successfully treated with this technique, eliminating the need for an autologous graft.^3^ Histological examination of the implanted matrices showed typical urethral stratified epithelium.^71^ Acellular collagen matrices have had poor results in patients needing tubularized graft, and in these patients collagen matrices seeded with cells demonstrated improved performance.^72^ In one observational study five boys with urethral stricture munderwent urethral biopsies, and muscle and epithelial cells were isolated and seeded onto tubularized synthetic scaffolds. The tissue-engineered tubularized urethra was then implanted into the patients. After 71 months median follow up, the urethras had normal function and anatomy. This technique represents a valid option in patients requiring difficult urethral reconstruction.^73^

Several studies have shown the feasibility of tissue engineering of the corpora cavernosa. Smooth muscle and endothelial cells seeded on collagen matrices have created a corpora cavernosa-like tissue structure in a rat model.^74^ Also, acellular collagen matrices derived from rabbit corpora and a biodegradable polymer scaffold were seeded with human corpus cavernosa muscle and endothelial cells. Using these scaffolds, Falke et al were able to form corporal tissue in vivo.^75,76^ Recently, neocorpora were engineered for total pendular penile corporal body replacement in a rabbit model. The bioengineered corpora were similar to the native tissue structurally and functionally.^77^ These results are promising for future patients with penile trauma.

Vaginal epithelial smooth muscle cells can be cultured in vitro^78^ and with cell seeded scaffolds, it is possible to engineer a vagina for total vaginal replacement.^79,80^

**Other Organs**

Stem cells play an important role in retinal regeneration. Studies have shown that stem cells have the potential to create a functional retina and form neural circuitry sufficient for vision.^81,82^ In one study ocular limbal cells cultured with keratinocyte stem cells from the corneal epithelium were grafted in 116 patients suffering from chemical destruction of limbus.^83^ The results suggest a promising potential for the restoration of vision in such patients. Regeneration of the orbital wall with autologous bone marrow stromal cells has also been demonstrated in a canine model.^84^ Regeneration of auricular cartilage, oral mucosa, tooth and periodontal tissue, and vocalcords are under investigation in several studies.^85,86,87,88,89^

## Conclusion

Trauma is an important health issue, particularly for young people needing replacement tissues or organs. Regenerative medicine has an essential role in the treatment of these patients. As discussed above, various tissues and organs are in different stages of investigation, with some already in clinical use and others in preclinical trials and experimental studies. As more products pass the FDA approval process and attain clinical applicability, regenerative medicine will hopefully be a valuable option for traumatic patients who need organ replacement and repair.
